# The complexity of *Rhipicephalus (Boophilus) microplus *genome characterised through detailed analysis of two BAC clones

**DOI:** 10.1186/1756-0500-4-254

**Published:** 2011-07-22

**Authors:** Paula M Moolhuijzen, Ala E Lew-Tabor, Jess A T Morgan, Manuel Rodriguez Valle, Daniel G Peterson, Scot E Dowd, Felix D Guerrero, Matthew I Bellgard, Rudi Appels

**Affiliations:** 1 Centre for Comparative Genomics, Murdoch University, South St., Perth, Western Australia, 6150, Australia; 2 Cooperative Research Centre for Beef Genetic Technologies, Armidale, NSW, Australia; 3 Queensland Alliance for Agriculture & Food Innovation, The University of Queensland, (c/o Department of Employment, Economic Development and Innovation), Locked Mail Bag No. 4, Moorooka, QLD 4105, Australia; 4 USDA-ARS, Knipling-Bushland U.S. Livestock Insects Research Laboratory, 2700 Fredericksburg Rd., Kerrville, TX 78028, USA; 5 Department of Plant & Soil Sciences and Life Sciences & Biotechnology Institute, Mississippi State University, 117 Dorman Hall, Box 9555, Mississippi State, MS 39762, USA; 6 Research and Testing Laboratory, 4321 Marsha Sharp Fwy, Lubbock, TX 79407, USA

## Abstract

**Background:**

*Rhipicephalus (Boophilus) microplus (Rmi) *a major cattle ectoparasite and tick borne disease vector, impacts on animal welfare and industry productivity. In arthropod research there is an absence of a complete Chelicerate genome, which includes ticks, mites, spiders, scorpions and crustaceans. Model arthropod genomes such as *Drosophila *and *Anopheles *are too taxonomically distant for a reference in tick genomic sequence analysis. This study focuses on the *de-novo *assembly of two *R. microplus *BAC sequences from the understudied *R microplus *genome. Based on available *R. microplus *sequenced resources and comparative analysis, tick genomic structure and functional predictions identify complex gene structures and genomic targets expressed during tick-cattle interaction.

**Results:**

In our BAC analyses we have assembled, using the correct positioning of BAC end sequences and transcript sequences, two challenging genomic regions. Cot DNA fractions compared to the BAC sequences confirmed a highly repetitive BAC sequence BM-012-E08 and a low repetitive BAC sequence BM-005-G14 which was gene rich and contained short interspersed elements (SINEs). Based directly on the BAC and Cot data comparisons, the genome wide frequency of the SINE Ruka element was estimated. Using a conservative approach to the assembly of the highly repetitive BM-012-E08, the sequence was de-convoluted into three repeat units, each unit containing an 18S, 5.8S and 28S ribosomal RNA (rRNA) encoding gene sequence (rDNA), related internal transcribed spacer and complex intergenic region.

In the low repetitive BM-005-G14, a novel gene complex was found between to 2 genes on the same strand. Nested in the second intron of a large 9 Kb *papilin *gene was a *helicase *gene. This *helicase *overlapped in two exonic regions with the *papilin*. Both these genes were shown expressed in different tick life stage important in ectoparasite interaction with the host. Tick specific sequence differences were also determined for the *papilin *gene and the protein binding sites of the 18S subunit in a comparison to *Bos taurus*.

**Conclusion:**

In the absence of a sequenced reference genome we have assembled two complex BAC sequences, characterised novel gene structure that was confirmed by gene expression and sequencing analyses. This is the first report to provide evidence for 2 eukaryotic genes with exon regions that overlap on the same strand, the first to describe *Rhipicephalinae papilin*, and the first to report the complete ribosomal DNA repeated unit sequence structure for ticks. The Cot data estimation of genome wide sequence frequency means this research will underpin future efforts for genome sequencing and assembly of the *R. microplus *genome.

## Background

The cattle tick, *Rhipicephalus (Boophilus) microplus (Rmi)*, is one of the most economically important ticks affecting the global cattle population [1]. Currently, *Rmi *and its associated pathogens can be transmitted to cattle and lead to severe agricultural losses in milk and beef production and restrict the movement of livestock. The most affected regions of the world are tropical and sub-tropical countries including northern Australia, Mexico, South America and South Africa, with threats to USA cattle populations at southern borders with Mexico [2].

The genome sizes of three species of ixodid ticks, *Ambylomma americanum *[3], *Boophilus *(*Rhipicephalus*) *microplus *and *Ixodes scapularis *(*Isc*) [4] have been estimated using Cot DNA reassociating kinetics, a procedure also used to estimate repetitive DNA in genomes [4]. The *Rmi *genome has an estimated size of 7.1 Gb, three times the size of the *Isc *genome (2.3 Gb) [4,5]. The *Rmi *genome is found to be composed of foldback (FB), highly repetitive (HR) and moderately repetitive (MR) elements, in the following proportion 0.82% FB, 31% HR, 38% MR, and 30% unique DNA, similar to *Isc *[4]. A short interspersed repetitive element (SINE) Ruka element, containing RNA polymerase III promoters, is major component of eukaryotic genomes that are particularly abundant in the heterochromatic compartment of vertebrates and plants as reviewed Kidwell and Sunter [6,7]. SINE transposable elements have the ability to move to new locations based on reverse transcription prior to genomic integration. Most SINEs are derived from tRNA [8], although some, such as the Alu family which accounts for approximately 10% of the human genome, are thought to originate from 7SL RNA sequences [9]. It has been shown in *R. appendiculatus *that secondary structure predictions indicate Ruka could adopt a tRNA structure similar to a serine tRNA [6].

The *Isc *Genome Project (IGP) [10,11], is the first tick genome sequencing effort and currently a major resource for tick comparative genomic analyses. This project has influenced the rapid rise in the number of sequences for tick DNA in NCBI [12]. The current *Isc *genome draft, represented by 369,492 supercontigs, (1.7 Gb) of linear genomic sequence was used in this analysis to identify conservation with available *Rmi *genomic DNA.

To provide insights into the complexity of the tick genome and that of specific BAC genes, the following *Rmi *sequence resources were available for analysis. The BmiGI Version 2 gene index [13] containing 13,643 non-redundant tentative consensus gene sequences. *Rmi *Cot reassociating kinetics genomic sequence, that has been demonstrated as a useful tool to explore the gene space of large genome species [14]. A BAC end library, created with the view to probe the *Rmi *genome for BAC sequencing [15]. A suppressive subtractive hybridization (SSH) to identify transcripts associated with host attachment and/or feeding, which identified both a large increase in rRNA transcripts thought to be associated increase protein production during tick feeding, and the production of a number of enzymes including serine protease inhibitors (Serpins) [16]. The results for these analyses are described.

## Results

### 

#### Selection of BAC clones for gene content: *Serpin *and *rRNA*

In order to select BAC clones for sequencing, BAC end sequences (BES) [17] were assessed against, the NCBI CDD [18], the BmiGI [13,19,20], and the SSH transcripts [16] (Additional file 1). The BAC clone BM-005-G14 (GenBank:HM748961) was identified in the BAC end analysis with significant alignment to a *serpin *conserved domain (CDD) [18] cd00172. The second BAC BM-012-E08 (GenBank:HM748964) was selected and sequenced based on significant alignment to *Rmi *EST sequence BEAE880F/R, a transcript highly expressed in tick responding to cattle [16].

The following result section describes the genomic; gene and comparative analyses for the BAC sequences BM-005-G14 and BM-012-E08.

### Analyses for BAC BM-005-G14: low repetitive, gene rich genomic region

#### BAC assembly and analysis

**Figure 1:**
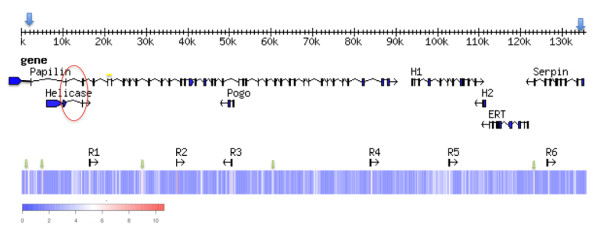
**BAC BM-004-G14 (135 Kb) Gene Structure and Cot sequence frequency**. Genes and Ruka elements (R1-R6) are displayed over a heatmap of Cot sequence frequency (log2), blue (low) = 0 and red (high) = 10, genes include a *papilin*, a nested *helicase*-like protein, a pogo element protein, two hypothetical proteins (H1 and H2), an endonuclease reverse transcriptase protein (ERT) and a *serpin*. Low complexity regions are shown over the heatmap (green arrow). Exon overlaps are circled in read.

The BAC clone BM-005-G14 was sequenced at 6.7× coverage (1,536 Sanger reads, insert size 135 Kb). The reads were *de novo *assembled with phred/phrap [21] into six contigs greater than 2 Kb and length 136,422 Kb. The BES positioning in two contigs confirmed the correct contig assembly. The final contig set was ordered and oriented by read linkage results, BES positioning and gene annotations. The BAC sequence was finished with gap closure into a 135 Kb genomic sequence (GenBank:HM748961). Gene prediction and comparative analysis identified regions of similarity to seven genes displayed in Figure 1. The forward strand contained: a *papilin *with a CDS length of 8,361 bp consisting of forty exons that span BAC sequence position 2,190 to 88,307 bp; a *helicase *with a CDS length of 4,800 bp consisting of four exons that span BAC sequence position 6,015 to 14,766 bp; a hypothetical protein (H1) with a CDS length of 2,394 bp consisting of eleven exons that span BAC sequence position 93,878 to 10,9076 bp. On the complementary strand; a pogo transposable element with a CDS length of 615 bp consisting of three exons that span BAC sequence position 49,728 to 50,977 bp; a hypothetical protein (H2) with a CDS length of 720 bp consisting of two exons that span BAC sequence position 110,728 to 111,698 bp; a hypothetical protein with a CDS length of 2,931 bp consisting of eleven exons that span BAC sequence position 112,452 to 122,035 bp. The hypothetical protein was conserved to *Isc *and similar to an endonuclease reverse transcriptase (ERT) in *Bos taurus*, the predicted CDS also contained a *serpin *domain (see later serine protease inhibitor result section). A final *serpin *with a CDS length of 2,766 bp consisting of ten exons that span BAC sequence position 123,297 to 133,688 bp (Figure 1).

Two genes of particular interest to the study were the serine protease inhibitor (*serpin*) (cd00172), originally targeted to select this BAC sequence, and the large multiple domain *papilin *gene spanning approximately 90 Kbp of the 135 Kb BM-005-G14 BAC sequence. The *papilin *an extracellular matrix glycoprotein that shares a conserved protein domain order in orthologous genes was then selected for further investigation.

#### *Papilin *and *Helicase *cDNA: resolving nested genes

A sequenced final *papilin *product of 8,761 bp, was merged from three cloned products, the 5' Race to primer AdamS_R1 product length of 867 bp, primer regions papilin57383F to PapilinR3 product length 7,723 bp and pap12440F to pap13230R direct sequence product length 813 bp (primers can be found in Additional file 2). The conserved domains are as follows: a Thrombospondin type 1 protein (TSP) domain (pfam00090) positioned 349-510 bp, an ADAM-TS Spacer 1 positioned 823-1167 bp (pfam05986), a set of four TSP domains in sequence positions 1204-1371,1387-1548,1561-1737,1724-1896 bp (pfam00090); ten BPT1/Kunitz family domains (KU) (cd00109) serine protease inhibitors can be found at positions 4654-4815,4831-4992, 5008-5169, 5185-5343, 5371-5532, 5545-5706, 5749-5907,5920-6081,6121-6279,6355-6510 bp; a whey acidic protein-type four-disulphide core domain (WAP) (pfam00095) in position 6901-7065 bp; a set of three immunoglobulin family (IG) (pfam07679) domains in positions 7198-7434, 7447-7680, 7864-8979; and a final protease and lacunin domain (PLAC) (pfam08686) positioned in the 8110-8208 bp region. Nested in intron 2 of the *papilin*, and on the same coding strand, is the *helicase*. This *helicase *gene overlapped exon regions with *papilin *exons 2 and 3 (Figure 1). *Helicase *exon 3 position 9,987-10,727 bp and *papilin *exon2 position 10,625-10,727 bp share 102 bp. The second shared exon region of 86 bp length was located between *helicase *exon4 position 14680-14766 bp and *papilin *exon3 position 14,680-14,813 bp. The shared overlap regions, circled in red in Figure 1, are shown in more detail in the sequence alignment, Additional file 3.

The expression of the *papilin *and *helicase *were determined in a number of tick life stages.

#### *Papilin *and *Helicase *qRT-PCR: gene expression in tick life stages

**Figure 2:**
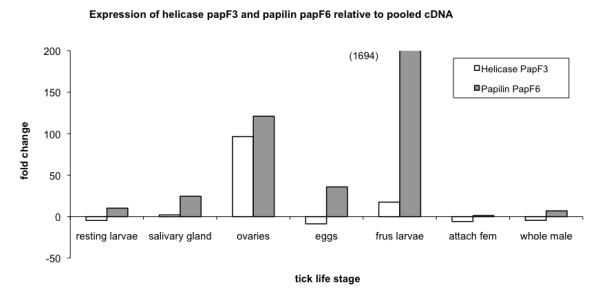
**Summary of *helicase *and *papilin *qRT-PCR fold change expression in tick life stages, relative to pooled samples, graph bars are *Helicase *(white) and *Papilin *(grey)**.

The gene expression fold change relative to pooled cDNA for a number of life stages were tested for both *papilin *and *helicase *genes. In quantitative real-time PCR (RT-PCR) analysis, it was demonstrated that expression of the *papilin *gene (white bar) was the strongest in tick larvae sensing and trying to attach to the host (Figure 2). The *helicase *(white bar) shows greatest up regulation in the ovaries of female ticks semi-engorged (17 days old) attach to the host. The *papilin *(grey bar) also showed differential up regulation in the ovaries. Confirming differential expression in at least two tick life stages tested.

As the *papilin *had increased expression in tick larvae sensing and trying to attach to the host, sequence level differences were examined between ixodid (tick) and mammalian (host) species for this gene, which is important in host and tick interaction.

#### Tick *papilin *comparative studies: Identifying tick-specific sequence differences

**Figure 3:**
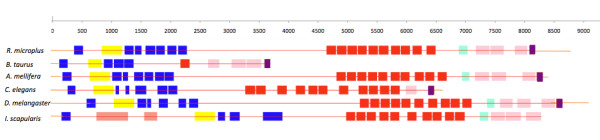
***Rmi papilin *(8761 bp) mRNA comparative domain analysis to *Bos taurus*(3794 bp),  *A. mellifera *(8424 bp), *C. elegans *(6654 bp), *D. melangaster *(9171 bp), and *I. scapularis *(8328 bp).** Types and number of domains are displayed, Thrombospondin (blue), Adam-TS (yellow), Kunitz (red), WAP (light blue), Ig-set (pink) and PLAC (purple).

**Figure 4:**
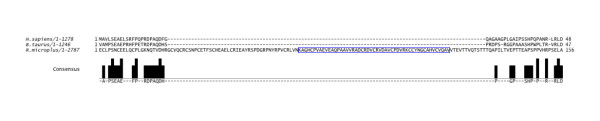
**Protein sequence alignment of WAP domain position, *Rmi (*2,180-2,335 bp), *Bos taurus *(963-1,009) and *Homo sapiens *(999-1,046) papilin protein alignment**. The *Rmi *WAP domain, position, 2,247-2,297 bp, (boxed light blue) in absent in mammalian sequences.

The mRNA sequence for *C. elegans *(NM_072616.3), *D. melanogaster *(NM_176574.2), *A. mellifera *(XM_396472.3), *I. scapularis *(preliminary data set cDNA jcvi 0.5 set 35859.m000024) and *B. taurus *(XM_002700672.1) was summarised to view domain differences, see Figure 3. The domain structure and number was closely conserved in the invertebrate species. Of interest however, the *helicase *domain nested in *Rmi *was also found in the *Isc*, the *papilin *sequence is not found in the later release 1.1. The number of conserved domains differed the greatest in *Bos taurus *(GenBank: XP_002700718.1) as compared to *Rmi*. The full *papilin *protein multiple sequence alignment between *R. microplus *and *Bos taurus *(XP_002700718.1) can be found in Additional file 4. These differences in domains include an extra full TSP domain and two fragments highlighted in blue in *Rmi*, a single bovine BTI/Kunitz serine protease inhibitor compared to the set of ten in *Rmi *(red) and the absence of a WAP domain upstream of the IG -set. The multiple protein sequence alignment of *Rmi *2,180-2,335 bp, human (sp|O95428.4) 999-1,046 bp and bovine 963-1,009 (XP_002700718.1) displays the WAP domain region, boxed in blue in *Rmi *(Figure 4) as absent in the mammalian sequences for *H. sapiens *and *B. taurus*.

**Figure 5:**
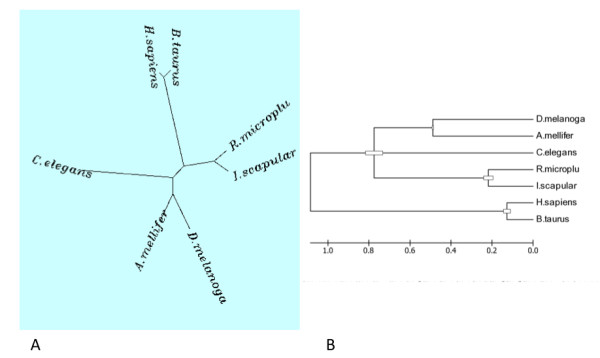
**Phylogenetic analysis of papilin protein for *Bos taurus *(XP_002700718**.**1), *Homo sapiens *(NP_775733.3), *Rmi , Isc *(35859.m000024_1), *D. melanogaster *(NP_788752.2), *Apis mellifera *(XP_396472.3) and *C. elegans *(NP_505017.1).** The trees are represented A) Neighbour-joining and B) Molecular clock.

Multiple sequence alignment [22] and phylogeny analysis [23] produced a mammalian clade for *Bos taurus *(XP_002700718.1) and *Homo sapiens *(NP_775733.3) and a tick clade for *Rmi *and *Isc *(35859.m000024_1), a hexapod clade for *D. melanogaster *(NP_788752.2) and *Apis mellifera *(XP_396472.3) and a single node for *C. elegans *(NP_505017.1) (Figure 5A). Evolutionary analyses shows that mammalian (host) *papilin *diverge at an earlier time than the divergence of hexapoda *papilin *from tick *papilin *(Figure 5B).

The *serpin *downstream of the *papilin *on the negative strand was investigated for gene synteny in other species.

#### Serine Protease Inhibitor: Serpin pseudogenes

Downstream of the *papilin*, a full CDS for *serpin *was predicted. The predicted *serpin *domain structure, however, was fragmented with the N-terminus and C-terminus rearranged, exon2 residues 266-364 and exon9 1-63 residues. Attempts to sequence the *serpin *cDNA resulted in a 500 bp product. A single PCR product based on forward primer (SerpF3) in exon5 and reverse primer (SerpR2) in exon9 was sequenced (Additional file 2). The small product sequenced matched only 148 bases of predicted exon5 and 231 bases of predicted exon9 (exons 6-8 were not in alignment). Conserved *serpin *domain analysis found also two fragments in predicted ERT gene exon2 residues 115-189 and exon4 residues 185-364. To determine whether the adjacent position of a *serpin *with *papilin *is common, a search of mosquito, fly genomes and *Isc *found no evidence of a *serpin *downstream from the *papilin *indicating this arrangement as not conserved within arthropods.

To gain better insight to genomic structures a Cot DNA comparison to the BAC sequence was undertaken.

#### BAC and Cot comparison: element genome wide frequency estimation

DNA reassociating kinetics based Cot filtration of genomic DNA was used to reduce the concentration of repetitive DNA sequences that dominate the *Rmi *genome, in order to analyse the "gene-rich" single/low-copy and the moderate repetitive DNA fractions [14,24]. Two fractions of moderate to low repetitive regions of *Rmi's *genome were selectively obtained from Cot filtration [14] and then analysed with BM-005-G14 BAC sequence, to assay the frequency of specific BAC sequence within the entire genome. Cot696 and Cot69 DNA 454 read sequences were mapped to BM-005-G14 to determine the frequency that the BM-005-G14 sequences were found in the Cot selected fraction of the tick genome. The read depth in a 100 bp window over total mapped read (bp) was calculated and the log2 value plotted as a heatmap (Figure 1). Six Sine *Ruka *elements [6] (R1-R6) were identified in BM-005-G14 (Figure 1) at positions 16,283-16,459, 37,204-37,398, 50,357-50,535, 83,819-83,996, 102,593-102,771, 126,277-126,452, the Pogo gene appear in the white-red bands, and (as expected) low frequency *papilin *and *serpin *in blue bands. Other regions of high frequency were identified as a 321 bp ATCT repeat positioned at 4,864-5186 and, a 124 bp TTTC repeat positioned at 878-1,004 bp and 241 bp CAA repeat in region 122,719-122,961, these are shown as green arrows over the heatmap in Figure 1. The overall estimate of BAC sequence coverage was 38.80% and 41.59% respectively for Cot 696 and Cot 69.

Based on the proportion of mapped reads relative to the total sequenced reads from the two Cot DNA experiments the genome wide frequency of a single 195 bp *Ruka *element (R2) at position 37,204-37,398 was estimated. The frequency of *Rmi Ruka *element in the genome was estimated based on the extrapolation of the two Cot fractions back to time zero to represent 0.42% (29 Mb) of the 7.2 Gb genome (Additional file 5). Although this estimate is approximate, the frequency of this specific *Rmi *Ruka element therefore in the genome is estimated to be at least 152,923 copies.

### Analysis for BAC BM-012-E08: highly repetitive genomic region

#### BAC assembly and analysis

The BAC clone BM-012-E08 was sequenced (1536 Sanger reads) at an expected size based on restriction digest of 65 Kb (not shown). Due to the complexity of BM-012-E08 BAC, the assembly metrics were tested to de-convolute the sequences (Additional file 6, 7). In summary, a conservative assembly approach [25] assembled less reads and produced more singletons but increased the length of the total assembly. BM-000-E08 was assembled into a 52 Kb consensus based on 18 contiguous sequences greater than 1 kb in size.

**Figure 6:**
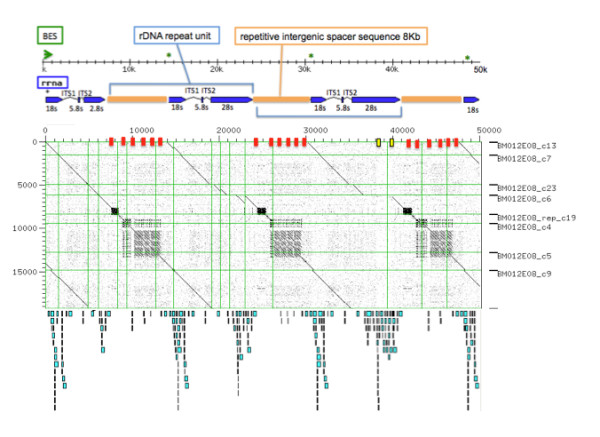
**BAC BM-012-E08 50 Kb sequence, with a repetitive 8 Kb intergenic spacer (brown), and rRNA 18S, ITS1, 5.8S, ITS2 and 28S (blue) together make the repeat unit structure (light blue)**. The BES start position are indicated by a green arrow and *copies. Below is BAC BM-012-E08 sequence dot matrix that displays the highly repetitive regions and rRNA (blue) fragments. On the horizontal axis regions confirmed (red) by PCR and not confirmed (yellow) are shown. Displayed below the dot matrix are the mapped Cot696 reads.

As very few arthropod retroelements and a large percentage of small RNA (17%) were identified with RepeatMasker [26], eight *de *novo interspersed repeat motifs were identified and masked for gene predictions. An almost complete, 18S ribosomal RNA gene, internal transcribed spacer 1 (ITS), 5.8S ribosomal RNA gene, ITS 2, and 28S ribosomal RNA gene, was identified by best sequence similarity to *Amblyomma americanum *(GenBank AF291874) (Additional file 6). In the assembly, there is evidence of at least three ribosomal 18S 5' and 28S 3' units. In Figure 6, the rDNA BAC positions are shown as blue arrows, BAC end positions in green, and the intergenic regions in brown. The remaining sequence not accounted for contained repetitive DNA sequence, similarly found in the highly repetitive intergenic regions between the repeating rDNA units. In Figure 6, the dot matrix of the best (parsimonious) ordered and oriented contigs display three rDNA units and repetitive intergenic regions aligned against a single unit.

The junction of the repeat motifs and the rRNA were tested by PCR, direct sequenced and clones sequenced to prove sequence and assembly accuracy. Three BAC repeat junction positions were confirmed by PCR and shown in Figure 6 as red markers on the horizontal axis of the dot plot (17 K, 22 K and 38 K). Primer sequences and gel pictures can be found in Additional file 2 and Additional files 8 and 9 respectively. In Additional file 8, lanes 1 and 2 confirmed marker 22 K downstream of the 28S unit in the large repeat region (dark blue glyph). Lanes 5 and 6 confirmed marker 38 K upstream of the 18S unit in the large repeat region. Marker 28 K confirmed downstream of the 28S unit in the smaller dense repeat region in contig6 Figure 6, this is repeated in contg-rep_c50 and contig1. The rDNA structure was further investigated.

#### Ribosomal DNA (rDNA) structure analysis

At least three full ribosomal 18S and 5.8S repeat units could be ordered although the 28S unit assembly was partial in the first repeating unit1, and in unit 3 contained a break point ITS (1 Kb) as compared to the *Ambylomma americanum *rRNA sequence. The rDNA repeat elements and ribosomal units were then tested by PCR to validate the gene size and to reveal more detail on the rDNA repeat unit and a putative interrupter sequence in LSU sequence that had previously been identified in the *Drosophila *genome [27]. Long range PCR confirmed rRNA unit size of 7-8 Kb (lane 2) and a large intergenic repetitive region of 8-9 Kb (lane 6) (Additional file 9). The 28S breakpoint/large interrupter region could not be confirmed by PCR, position is highlighted yellow in Figure 6 (~42 Kb), tested primer sets 15 K and 18 K can be found in Additional file 2. The BAC assembly and long range PCR confirmed the rRNA unit size of 8 Kb and a large intergenic repetitive region of at least 9 Kb. The *Rmi *18S sequences were then analysed for tick specific differences.

#### Tick ribosomal DNA comparative studies: Identifying tick-specific sequence differences

To identify sequence differences in rRNA protein binding sites the 18S small subunit (SSU) was aligned to the 16S *E. coli *SSU and bovine18S.

**Figure 7:**
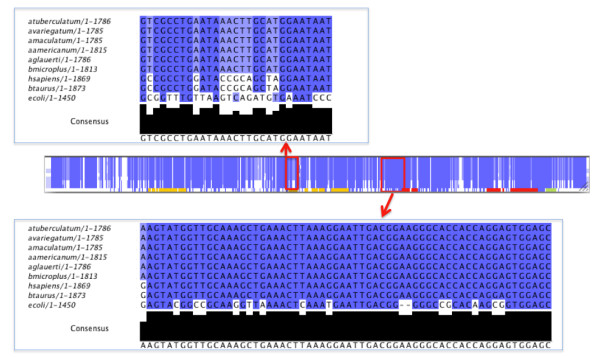
**Overview of multiple sequence alignment; six tick species 18S *A. americanum, A. glauerti, A. variegatum, A. tuberculatum, A. maculatum *and *R. microplus*; two mammalian species 18S tick host *B. taurus *and *H. sapiens*, and *E. coli *16S**. *E. coli *16S protein binding sites are highlighted S7_S9_S19 complex (red), S8_S15_S17 complex (orange), S8_S17 complex (green) (Weiner et al 1988). Top figure shows multiple sequence alignment with binding site of sequence differences (*Rmi *791-818 bp). Bottom figure shows multiple sequence alignment with tick/mammalian conserved SNP *Rmi *1122-1160 bp.

Analysis of three *E. coli *complex binding sites found a number of sites with conserved changes in tick as compared to mammals. Sites were also identified conserved in *E. coli *and mammals, and a conserved change found in tick (Figure 7).

In Figure 7, regions for rRNA protein binding [28] have been highlighted to identify regions of differences in *E. coli *16S (Embl: X80725) and tick and mammalian 18S sequences. Represented in the multiple sequence alignment are five tick species, *A. americanum *(GenBank:AF291874.1), *A. glauerti *(GenBank:AF115372.1), *A. variegatum *(GenBank:L76346.1), *A. tuberculatum *(GenBank:L76345.1), *A. maculatum *(GenBank:L76344.1) and *R. microplus *18S RNAmmer prediction [29], the ticks' hosts *B. taurus *(GenBank:DQ222453.1) and *H. sapiens *(GenBank:NR_003286.2). In *E. coli *16S protein binding site complexes are highlighted in the following *E. coli *sequence regions: S7_S9_S19 complex (red) 936-965, 972-991, 1208-1262, and 1285-1379 bp; S8_S15_S17 complex (orange) 118-240, 576-606, 629-685 and 706-769; S8_S17 complex (green) 1406-1442 and 1462-1494 bp (Weiner et al 1988). The multiple sequence alignment and annotation can be found in Additional file 10. The protein rRNA binding site for protein S7 in *E. coli *(1236-1240, 1373-1383 bp) shows a single SNP between the tick and mammalian sequences. In Figure 7 the top blue boxed alignment subset shows a highly conserved region between 18S and 16S sequences (*R. microplus *1122-1160 bp) with an example conserved SNP change G- > A in tick, this was confirmed in all three 18 s units. A total of 3.2% (59) of *Rmi *18S sequence were conserved SNPs. The lower boxed alignment subset (*R. microplus *791-818 bp) shows more differences in the conservation with the S8_S15_S17 binding complex in *E. coli*. In 18S sequences only, a total of 63 *Rmi *positions showed conservation in all tick species and a conserved change in *B. taurus *and *H. sapiens*.

To examine if this BAC sequence had the same variation at the R2 retrotransposon target site of the LSU [30], the *Rmi *rDNA LSU positioned at 19240-23971 bp (19 K unit) and 35316-40798 bp (35 K unit) were aligned. The specific variation of conserved SNPs guanine (G) and thymine (T) previously found for *Ixodidae *(hardback ticks) were confirmed, at positions 2,800 bp (G) and 2,801 bp (T) for the 19 K unit and 2,873 bp (G) and 2,874 bp (T) for the 35 K unit. No R2 retroelements were identified, a small fragment of LINE/R1 element TRAS9_SC was however found in the 35 K unit at position 1,801-1,889 bp.

#### BAC and Cot comparison: element genome wide frequency estimation

To estimate the genome wide frequency of elements in this highly repetitive BAC, a single Cot fraction Cot 696 was used to align to BM-012-E08. The reads were mapped to the BAC sequence at 100% identity and 90% read coverage, shown beneath the dot matrix in Figure 6. Reads could not be aligned to the densely repetitive rDNA intergenic regions, it was also noted that the short interspersed Ruka element found in BM-005-G14 was absent in BM-012-E08.

## Discussion

In the absence of a reference genome we describe the de novo assembly and in-depth analysis of two *Rmi *BAC clones, selected for the following two reasons. The first for gene content, based on *Rmi *expression analyses identified as important during feeding and protein production during fast growth stages of tick development. The second reason was for BAC clones with different genomic complexity, to make Cot DNA comparisons. In this study of two very different BAC regions newly reported features for eukaryotes and *Chelicerate *genomes are described.

The following discussion sections address the study of both the selected BAC sequences.

### Tick genomic structure: assembly and predictive models

The correct assembly of tick genome is a challenge due its repetitive nature, and the lack of predictive models for gene structures. The BAC assembly, due to low genome level synteny with the *Ixodes scapularis *assembly, depended on the comparative analysis of transcript and the positioning of BES.

The correct assembly of the BM-005-G14 contig set was dependant on the correct BES positioning, while the ordering and orientation was aided by the BES positioning and transcript alignments.

Given the complexity of BM-012-E08, different assembly tools were trialled under different options. Assembly tools with uniform read distribution take a cautionary approach in contig building, and sometimes create two contigs when it could have created one. This feature reduces over compression of repeats during the contig building phase and ensures that, for example rRNA stretches which are present multiple times in a the genome will also be present approximately the same number of times in the result files [25]. The repeat motifs, intergenic regions, rRNA sequences and rDNA unit size were confirmed by PCR. However the assembly gaps and insertions show clear deviations from the perfect repeat unit size. This is the first *Rhipicephalinae *assembly of rDNA and the first known attempt at assembly in *Arthopoda *of three external intergenic repetitive units between the rDNA repeating subunits.

### Tick gene structure: predictive models

The complexities of gene predictions included intronic regions of nested repeat elements, multiple short exons and overlapping regions complicating the delineation of exon coding regions. Overlapping genes have been reported in *Drosophila *but these genes were on different stands [31]. In eukaryotic research this is the first description of same strand gene overlap between two genes, the *papilin *and *helicase*.

In *Drosophila, papilin *an extracellular matrix glycoprotein is found to be involved in, (1) thin matrix layers during gastrulation, (2) matrix associated with wandering, phagocytic hemocytes, (3) basement membranes and (4) space-filling matrix during *Drosophila *development [32]. Essential also for normal embryonic development *Caenorhabditis elegans *[33]. This is also the first *Chelicerate *full-length *papilin *cDNA sequence produced. Our *papilin *gene model (refer to methods) was confirmed by other arthropod species. The *papilin *nested *helicase *was also found within the *Isc *genome supercontigs (version 1), this inclusion of the *helicase *shows a level of gene synteny is present in this region between the two distant hard tick species.

It has been reported that inhibiting *papilin *synthesis in *Drosophila *or *Caenorhabditis *causes defective cell arrangements and embryonic death. Ectopic expression of *papilin *in *Drosophila *causes lethal abnormalities in muscle, Malpighian tubule and trachea formation. It has been suggested that *papilin *influences cell rearrangements and may modulate metalloproteinases during organogenesis [32-35].

These function/activities relate to the following specific domains. An interesting gene domain complex the tick derived Kunitz type inhibitors act as antihemostatic factors [36]. Hematophagous organisms must overcome host hemostasis in order to locate blood and maintain its flow during ingestion [37]. Salivary components produce antihemostatic, anti-inflammatory, and immunosuppressive effects that may facilitate feeding, as well as transmission of tick-borne pathogens [37]. The number of *Rmi *KU domains (x10) present compared to bovine (x1) indicates, based on this domains function, an important change in this genes structure for tick survival.

The whey acidic protein-type four-disulphide core domain (WAP) has protein family members that include the whey acidic protein, elafin (elastase-specific inhibitor) known to have anti-microbial acitivity [38], catrin-like protein (a calcium transport inhibitor and other extracellular proteinase inhibitors. A significant sequence variance in bovine was the absence of the WAP domain (Figure 4).

Isoforms of *papilin *have been found in a number of *Arthropoda *species, six in *Drosophila *and two in *Apis*. Given the size and complexity of the *Rmi papilin*, isoforms may exist that are yet to be investigated.

The *helicase *was identified nested as a separate gene between the first 5' thrombospondin and the Adam-TS spacer of the *Rmi papilin*. RACE sequencing from the Adam-TS spacer domain exon in the 5'end direction produced the complete *papilin *product minus the *helicase *insertion, confirming our gene model.

The discovery of shared exon regions for 2 eukaryote genes, the *papilin *and *helicase*, is quite novel. Nested genes do occur in eukaryotes [31], nested genes in *D. melanogaster *and *C. elegans *have been found exclusively as embedded sequences in introns. Kumar 2009, reviewed that in *D. melanogaster *nested intronic genes constitutes approximately 6% of the organism's total gene complement, and 85% of these nested genes are predicted to encode protein. For example the *gart *locus, the *Pcp *gene is nested in intron 1 of the *ade3 *gene on the complement strand. A nested ketoreductase was identified in an *A. aegypti *papilin - however not with exon overlap as shown for the *helicase *identified here. In the mouse genome, 28 overlapping gene pairs had partial overlapping exons, and did not encompass the entire coding sequence of either gene. In the human genome 51 exon overlaps on opposite strands, again were partial. Neither the human nor the mouse genome contains any overlapping genes that share coding sequences on the same strand. Further the majority of nested intronic genes are functionally unrelated and typically not co-expressed with their external host genes. Therefore further functional analysis of this gene's novel arrangement warrants investigation. No *helicase *element was found nested in the bovine intron region of *papilin*.

The initial identification of the *serpin *domain led to the adjacent *papilin *gene described above. No syntenic evidence was found for the down stream serpin region in *Isc*. Full investigation of this gene family within *Rmi *genomic sequence and the *I. scapularis *genome remains to be investigated.

The genes for ribosomal DNA are tandem repeated clusters in the heterochromatic regions of metazoan genomes [27,39], in *Drosophila *77% of heterochromatin sequence is composed of fragmented and nested transposable elements and other repeated DNAs [39]. It has been reported in vertebrate the splitting and apparent splicing of ribosomal RNA occurs, and during processing, in mammalian nuclear 28S pre-rRNA, tissue-specific elimination of an 'intron' bearing a hidden break site occurs [40,41]. An almost complete, 18S ribosomal RNA gene, internal transcribed spacer (ITS) 1, 5.8S ribosomal RNA gene, ITS 2, and 28S ribosomal RNA gene, was identified by sequence similarity to *Amblyomma americanum *(GenBank AF291874; [30]), which is the only other tick rDNA sequence analysed at this level of coverage. A similar unit was not identified in the *Ixodes scapularis *genome highlighting the difficulty in the assembly of this region. The *Rmi *rDNA units have been identified as novel due to the lack of the R2 retroelement previously identified in *Amblyomma *and now confirmed in this study, even though the R2 retroelement binding site hard tick sequence difference was conserved. The fragmented nature of the LSU makes it possible that the BM-012-E08 BAC clones are derived from the end of an array of rDNA units in the genome where incomplete and rearranged rDNA units may occur.

### Tick DNA comparative studies: Identifying tick-specific sequence differences

A number of conserved changes within rRNA protein binding sites between the ticks as compared to mammals were found. The hard tick specific sequence differences (SNPs) were also found in the LSU R2 retroelement target site.

Due to the uniqueness of tick rDNA sequences it is feasible that tick rRNA could be a target for drug development analogous to the use of bacterial rRNA as antibiotic targets. Consistent with this possibility, a number of conserved changes within rRNA protein binding sites between the tick as compared to mammal were found. The hard tick specific sequence differences (SNPs), were also found in the LSU R2 retroelement target site.

### Tick gene expression analysis

The qRT-PCR analysis of the *papilin *described confirmed differential increased expression in two life stages, most prominently in larvae trying to attach to the host (Figure 2). It was also demonstrated that the *helicase *is strongly expressed in ovaries of feeding females. *Helicases *are often utilized to separate strands of a DNA double helix or a self-annealed RNA molecule using the energy from ATP hydrolysis, a process characterized by the breaking of hydrogen bonds between annealed nucleotide bases. The differential expression of both the *papilin *and *helicase *in adult female ovaries suggests that is perhaps a conserved functional arrangement.

The abundance of RNA proteins as identified in the subtraction library study was not surprising due to increased protein production during feeding. Anderson et al. 2008 reported that the abundance ribosomal protein coding genes is not unusual for a transcriptome analysis and illustrates the high degree of redundancy found in such libraries, especially the occurrence of numerous sequences coding for proteins involved in protein synthesis such as ribosomal RNA, e.g. 40S, 60S and other ribosomal genes [42].

### The analysis of genome sequence via back end sequencing and Cot DNA

The *Rmi *genomic DNA that was enriched for single/low-copy and moderately repetitive DNAs [14] along with BAC end sequencing have provided valuable insights into *Rmi *genomic structure. Mapping the reads of the Cot DNA to the BAC sequencing identified regions of high repetitive content in BM-012-E08 complex intergenic region by the absence of mapped reads. Also moderately repetitive regions could be identified such as the RUKA element in BM-005-G14.

In particular using the two Cot filtrations, we were able to estimate the frequency of any specific genomic sequence within the entire genome. As an example presented, major frequency peaks were identifiable and the relative frequency of the sine Ruka [6] element in the genome was estimated for BM-005-G14. Although absent from the euchromatic section of the relatively compact 1.8 Gb genome of *Drosophila melanogaster *[43], several distinct families of SINEs with copy numbers of up to 590 Kb per genome have been described in *Aedes aeygpti*, the mosquito vector of the yellow fever virus [44,45]

The frequency of a single *Rmi *Ruka element in the genome was estimated based on the extrapolation of the two Cot fractions to represent 0.42% of the genome, at least 152,923 copies.

A previous examination of 3 BAC sequences, and the (DFCI) Gene Indices [46] for the four ixodid tick species, *A. variegatum *[47], *R. appendiculatus *(*Rap*) [48], *B. (R.) microplus *[19] and *I. scapularis *[49], estimated that the Ruka repeat sequences comprise approximately 1.6% (4 kb) of the 250 Kb of *Rap *genome (BAC sequence). Then on the following assumptions (1) that these *Rap *BAC contigs are representative, and (2) a genome size for *R. appendiculatus *of 1 Gb, that a total of 65,000 copies of Ruka could be predicted [6]. Since our estimation in *Rmi *is based on a single element we expect the number of Ruka families will occupy a much larger fraction of the genome than previously estimated.

## Conclusion

This analysis builds on the previous report by Guerrero et al 2010, to characterise genomic DNA in the tick *Rhipicelphalus microplus*, The complete secreted extracellular matrix protein gene *papilin *primarily found in basement membranes and essential for embryonic development, was assembled and cDNA sequenced. This is the first reporting in eukaryotes of same strand exon overlap between the sequenced products of *papilin *and *helicase*. Detection of these types of overlaps is a complication for current de-novo gene prediction tools. In a second BAC clone, ribosomal DNA (rDNA) was assembled into three repeat units, the first rRNA assembly in *Rhipicephalinae*, and the first attempt to assemble sequence of the rDNA repeat units and intergenic spacer in arthropods.

In both *papilin *and rRNA, tick specific sites of sequence variation were identified in tick *R. microplus *relative to the host *Bos taurus*, in a detailed comparison to identify targets for disrupting the pathogen-host interaction. In addition expression analysis of *papilin *and *helicase *demonstrated striking tissue specific expression in response to sensing the host prior to attachment for feeding.

Finally the two Cot-filtration resources provided a means to estimate the frequency of an element in the context of the whole genome.

In order to place the BAC sequences into a whole genome context, the BAC sequences were probed with 454 sequenced Cot 69.56 secs and 696.6 secs DNA. This analysis allowed the representation of specific BAC sequence to be estimated within the respective Cot DNA sequences and thus estimate the frequency of sequence occurrence in the whole *R. microplus *genome.

The BAC, BAC end sequences (BES) and Cot DNA have allowed an in-depth analysis of selected *R. microplus *genomic DNA, and in terms of sequencing towards a whole genome provided a valuable insight into *R. microplus *genomic structure.

## Methods

### BAC end sequences

Glycerol plates with BAC clones (1,125 96-well plates) were submitted to Beckman Coulter Genomics (Beverley, MA, USA) to obtain approximately 12,000 reads using bi-directional sequencing of the clones. The Beckman Coulter Genomics protocol is described as follows: clones were picked from the 96 well plates, cultured and DNA was purified using SPRI^®^; following dye-terminator fluorescent sequencing the product was purified using CleanSEQ^® ^with sequencing fragments detected via ABI3730xl capillary electrophoresis. The total 10,582 BAC end sequences (BES) provided as trace files from Agencourt were clipped of the vector (pECBAC1) with cross_match, Phrap package version 0.990329 [21]. Sequences greater than 500 bp have been deposited GenBank GSS under HN108288-HN118367.

### BAC Genomic DNA extraction, library construction, and BAC screening and sequencing

Ticks from the Deutsch strain of *R. microplus *were reared at the USDA-ARS Cattle Fever Tick Research Laboratory in Mission, TX [50]. Genomic DNA extraction, library construction, and BAC screening are as described by Guerrero et al., [14].

### BAC sequencing

BAC vector used was pECBAC1 and the cloning site BamHI. BAC libraries were sequenced using 3-4 kb insert high copy shotgun library methods aiming for 8-fold coverage of 1,008,000 bases (high copy) using Sanger Sequencing ABI technology as described for the BAC end sequencing above (Beckman Coulter Genomics, MA, USA).

phred 20 read lengths greater than 700 bp and pass rates: > 90% and x6 coverage.

### BAC assembly

BAC Sanger assemblies were conducted with phred/phrap [51], CAP3 [52] and Phusion [53] and MIRA [25]. The BES were mapped to the assembly with BLAT [54] at 100% percent sequence identity. Dot plot matrices were generated using Dotter64 [55]. Beckman Coulter Genomics (MA, USA) closed the sequence gaps based on pair end read linkage. The following BAC sequences have been deposited in GenBank, BM-005-G14 (HM748961) and BM-012-E08 (HM748964).

The correct orientation and ordering of the contigs was based on pair-end read assembly linkage results, back end sequencing positioning and gene annotation, as comparative analysis of *Rmi papilin *to a number of species show ordered domain conservation. The finished BAC with gap closure was 135 Kb, close to the estimated restriction digest size, with the *papilins' *coding region of 8 Kbp spanning a genomic region of 86 Kbp. The *papilin *gene was first predicted with Genscan HumanIso model from the 2 large coding sequences CDS8 and CDS6 (6,663 and 4,077 bp respectively), which covered all the *papilin *protein domains, except the initial 5' end thombospondin domain. In addition, CDS8 contained a helicase domain (not previously identified in *papilin*), an Adam-TS-spacer 1 and the second expected thombospondin domain. CDS6 contained all the remaining *papilin *domains, KU domains x10, WAP, IG x3, and the final 3'end PLAC domain. Direct cDNA sequencing confirmed the BAC data and 5' RACE assisted to confirm the presence of the missing thrombospondin domain and our model subsequently proposed the presence of 2 genes, the *papilin *and the *helicase*.

### BES analyses

BlastN [56] nucleotide similarity searches were conducted on Dana Faber Cancer Institute (DFCI) Gene Indices BmiGI [13,19], IscGI [49], subtraction library cDNA [16] and iscapularis.preliminary.TRANSCRIPTS_JCVI-IscaW1.0.5. and NCBI [57] datasets that included, (nr, est, genomic, refseq, GSS, WGS).

BlastX [56] protein similarity searches were conducted on NCBI [57] (nr, patent), and *Ixodes scapularis *peptide gene predictions 1.1 iscapularis. PEPTIDES-IscaW1.1 protein datasets.

Domain and protein family identification was conducted with RPSBlast, on NCBI Conserved Domain Database (CDD) [18] database.

BLAT [54] ixodes_scapularis_supercontigs.

### Gene prediction

Genscan [58] (model for human isoforms) was used to assemble the BAC contigs. Bioperl [59] scripts were used to parse alignments and identify conserved regions. The In-silico workflow was designed based on open source applications and CCG Grid computing [60].

### Sequence alignment and phylogeny

ClustalW [22] was used for multiple sequence alignments and multiple sequence alignments for manuscript were displayed in Jalview [61].

Phylogeny analysis was conducted with Phylip version 3.6 [23] protein distance algorithm and Neighbor-Joining method [62] and bootstrap test of 100 replicates.

The molecular clock test was performed by comparing the ML value for the given topology with and without the molecular clock constraints under Jones-Taylor-Thornton (1992) model [63,64]. Evolutionary analyses were conducted in MEGA4 [64].

The evolutionary history was inferred using the Neighbor-Joining method [62]. The bootstrap consensus tree inferred from 500 replicates was taken to represent the evolutionary history of the taxa analyzed. The percentage of replicate trees in which the associated taxa clustered together in the bootstrap test (500 replicates) are shown next to the branches. The tree is drawn to scale, with branch lengths in the same units as those of the evolutionary distances used to infer the phylogenetic tree. The evolutionary distances were computed using the method [65] and are in the units of the number of amino acid differences per site. The analysis involved 7 amino acid sequences. All positions containing gaps and missing data were eliminated. There were a total of 1124 positions in the final dataset.

### Repeat identification

Arthropod known repeats were identified with RepeatMasker version 3.2.6 [26]. Repeatscout [66] was used for the *de-novo *identification of repeat motifs. Perfect tandem repeats were identified with SSR finder Perl program. Sine RUKA elements were identified based on BlastN [56] homology to GenBank: EU018139.1 (9,947-10,084 bp), percentage identity greater than 84% and coverage greater than 69%.

### cDNA preparation

Total RNA was extracted from tick samples using Trizol reagent (Invitrogen Corporation, CA, USA). Tissue was ground to a fine powder using a mortar and pestle with liquid nitrogen and the powder transferred to a tube of Trizol with 1 mm glass beads. This mix was further homogenised for 45 seconds in a MiniBeadbeater-96 (Biospec Products, Bartlesville, OK, USA) then the RNA was extracted using chloroform and isopropanol. Double stranded cDNA was created from 25 μg of total RNA using a SuperScript™ Double-Stranded cDNA Synthesis Kit following Kit protocols (Invitrogen Corporation, CA, USA).

### *Papilin *PCR amplification and sequencing

Primers based on BAC sequences were designed with EMBOSS eprimer3 [67] and a minimum GC clamp of 2. Synthesis of primer sequences were by Sigma Aldrich (MO, USA) and sequences are presented in Additional file 2. The full *papilin *cDNA was PCR amplified from cDNA extracted from frustrated larvew and cloned in three steps. A 7723 bp product was amplified between primers Papilin57383F and PapilinR3 (designed from predicted coding sequence) using the Expand Long Template PCR System (Roche Applied Science, Mannheim, Germany) using Expand Long Template Buffer 2. This reaction was thermocycled in a DNA Engine (PTC-200) Peltier Thermal Cycler (Biorad Laboratories, CA, USA). The purified product was transformed into chemically competent One Shot^® ^TOP10 cells using a TOPO-XL^® ^PCR Cloning Kit (Invitrogen Corporation, CA, USA). For each transformation, DNA was prepared from six clones using a QIAprep Spin Miniprep Kit (Qiagen, CA, USA). Plasmid inserts were sequenced using Big Dye Vers 3.1 technology (Applied Biosystems, CA, USA) and were run on an Applied Biosystems 3130xl Genetic Analyser (Griffith University DNA Sequencing Facility, School of Biomolecular and Biomedical Science, Griffith University, Qld, Australia). Sequences were edited and aligned in Sequencher (Vers 4.8 Gene Codes Corporation, Ann Arbor, MI, USA). Additional sequencing primers were designed manually (Additional file 2).

The start codon for the papilin gene was determined following 5' amplification of the cDNA ends from larval cDNA using the SMARTer™ RACE cDNA Amplification Kit as described by the kit manufacturer (Clontech Laboratories Inc., CA, USA). The 5'-RACE PCR used an Advantage 2 PCR kit (Clontech Laboratories Inc., CA, USA) using the kit 5' RACE UPM primer and the gene specific reverse primer AdamSR1 designed within the Adam spacer region of the papilin gene (Additional file 2). The gel-purified product was cloned into chemically competent One Shot^® ^TOP10 cells using a TOPO^® ^TA Cloning Kit (Invitrogen Corporation, CA, USA). Clone inserts were sequenced as described above.

The *papilin *stop codon was determined from the predicted coding sequence and a primer was designed anchored at the stop position PapStopR1 (Sup). A 611 bp product was PCR amplified between primers Pap12440F to PapStopR1. The product was cloned and sequenced as described above.

### *Papilin *cloned products

Final *papilin *product was 8,761 bp, from the 5' Race to primer AdamS_R1 product length 867 bp, and from primer regions papilin57383F to PapilinR3 product length 7,723 bp and pap12440F to pap13230R direct sequence.

### *Helicase *PCR amplification and sequencing

A second large PCR product 4886 bp in length was amplified from the larval cDNA between primers PapilinORFF2 and Papilin54900R. The 3' end of the product has a 229 bp overlap with the papilin gene (exon 2 and 3, 5' of the Adam spacer). The product was amplified using the Expand Long Template PCR System (Roche Applied Science, Mannheim, Germany) and was cloned and sequenced as described above for the large papilin clone. Internal primers were designed to sequence the complete clone that was found to contain a helicase gene (Additional file 2).

### BM-12-E08 PCR

PCR 50 μl reaction contained Advantage 2 SA PCR buffer, 10 mM of dNTP mix, 10 μM of each primer, 100 ng of DNA template and Advantage 2 polymerase mix as recommended by the manufacturer (Clontech Laboratories Inc., CA, USA), Cycling Parameters (BIORAD DNA Engine Cycler): Initial denaturation for 2 mins at 94°C followed by 29 cycles of denaturation 1 min 94°C, annealing 1 min 55°C, and extension 1 min 72°C, with a final extension of 7 mins 72°C. The products were visualised following agarose gel electrophoresis (1.2%) containing Gel Red (Jomar Bioscience Pty Ltd, SA, Australia). PCR products and purified plasmid DNA were sequenced using Big Dye Vers 3.1 technology (Applied Biosystems, CA, USA) and were run on an Applied Biosystems 3130xl Genetic Analyser at Griffith University DNA Sequencing Facility (GUDSF). Sequences were edited and aligned in Sequencher (Vers 4.7 Gene Codes Corporation).

Amplified products were cloned using the pCR2.1 - TOPO plasmid vector (Invitrogen Corporation, CA, USA). Transformed cells were plated on to LB agarose plates containing 50 μg/ml kanamycin and grown overnight at 37°C. Colonies were picked and cultured in LB medium broth containing 50 μg/ml kanamycin. PCR reactions were performed on 1 μl of the cultured broths and analysed by agarose gel electrophoresis to confirm insertion. Plasmid DNA was purified as described above.

### BM012-E08 Long range PCR

Tick genomic DNA was prepared following tissue grinding as described above for cDNA preparation and subsequently purified using the QIAamp DNA mini-kit as described by the manufacture (QIAGEN, CA, USA). The Expand Long Template PCR system was used to amplify the DNA under conditions recommended by the manufacturer (Roche Applied Science, Mannheim, Germany) in a BioRad DNA Engine Peltier Thermal Cycler. Direct sequencing was undertaken as described above. All sequence alignment graphs were generated with Bioperl [59].

### qRT-PCR analysis

Primers were designed manually within targeted exon regions for the *helicase *and *papilin *transcripts described in this study (Additional file 2). Methods for qRT-PCR analysis were described previously by Lew-Tabor et al. (2010), utilising tick extracts prepared from different tick organs and stages, normalised against 2 housekeeper genes (*Actin *and rRNA 18S) and against a pooled cDNA sample.

### Cot selected genomic DNA

The Cot filtration on *Rmi *genomic DNA was performed as previously described [14], to enrich for single/low-copy and moderately repetitive DNAs. The two "conditions" are called Cot69 and Cot696. Starting DNA concentrations for both were 200 micrograms of sheared genomic DNA. Time for renaturation was 1 hr, 48 min, 6 sec for sample Cot696 (Cot of 695.6) and 10 min 49 sec for sample Cot69 (Cot of 69.56). Renaturation was conducted at 70 degrees C, at 0.03 M NaPO4. Sequencing results form these two Cot-selected samples have been deposited in GenBank SRA, submission: SRA012677.4/SID00001.

Cot DNA 454 read sequence was mapped to the BAC sequence using the Newbler GS-FLX reference mapper, version 2.0.00.20 [68] and BLAT [54]. The total read number was calculated for each BAC window size 100 bps (read number per 100 bp window). The percentage (read number per 100 bp window)/(Total BAC read count) was then plotted for each BAC. The gnuplots were calculated by (Sum bp depth/window size)/(Sum total bp depth/BAC length).

## Competing interests

The authors declare that they have no competing interests.

## Authors' contributions

PM carried out the bioinformatics analysis, and drafted the manuscript. RA, MB, ALT, FG, MRV, JATM participated in the interpretation of analysis and writing/editing of this manuscript. FG (USDA) funded the Cot filtration and sequencing. FG performed BAC library filter hybridizations and the Cot selection protocols, and participated in the selection of BAC sequences. ALT (BeefCRC) funded the sequencing of the BES and BACs, and supervised PCR and cDNA sequencing. JATM cloned and sequenced *papilin *and *helicase *genes and conducted qRT-PCR analyses. DP participated in and supervised the Cot-selection protocols. SD performed the 454 sequencing of Co-selected DNA. RA supervised the design and analysis for this manuscript. All authors have read and approved the final manuscript.

## Supplementary Material

Additional file 1 **BAC end sequence analysis**. Results for BAC end sequence database searches. Click here for file

Additional file 2 ***Rmi *primer sequences and positions**. Primer sequence and sequence positions for BAC sequences BM-012-E08 and BM-004-G14 (*papilin *and *serpin*). Click here for file

Additional file 3 **Sequence alignment of exon overlap between *papilin *and *helicase***. Sequence alignment of exon overlap between *papilin *position 502-738 bp and *helicase *3 positions 974-4802 bp. The consensus black bar indicates the region of overlap. Blue arrows indicate exon junctions, *papilin *T-G positions 604 and 605 and *helicase *G-C positions 4715, 4716. Click here for file

Additional file 4 ***Rmi *and *Bos taurus papilin *protein sequence alignment**. *Rmi *and *Bos taurus papilin *protein sequence alignment, *R. microplus *and *B. taurus*. Domains are highlighted Kunitz BPTI (red), ADAM spacer1 (yellow), Ig-set (pink), PLAC (purple), WAP (orange) and TSP1 (blue). Click here for file

Additional file 5 **BM-005-G14 Ruka frequency analysis**. Table and graph of BM-005-G14 Ruka frequency analysis using two Cot DNA experiments. Click here for file

Additional file 6 **BAC BM-012-E08 sequence assembly statistics**. Table of BAC sequence assembly statistics BM-012-E08. Click here for file

Additional file 7 **BM-012-E08 sequence dot matrix with *Ambylomma *rRNA alignment**. Figure of full dot matrix BM-012-E08 with *Ambylomma *rRNA (blue) alignment. Click here for file

Additional file 8 **BM-012-E08 repetitive elements PCR results**. PCR results for BM-012-E08 repetitive elements, lanes: 1) 22900 (F1/R1) 2) 22900 (F2/R2) 3) 17000 (F1/R1) 4) 17000 (F2/R2) 5) 38000 (F1/F2) 6) 38000 (F2/R2) 7) negative control. Click here for file

Additional file 9 **BM-012-E08 long primer sets to amplify tick genomic DNA**. Long primer sets used to amplify tick genomic DNA using Roche Expand Long Template PCR system, Lane 1 Fermentas Mass ruler 80 bp-10 kb (#SM0403), Lane 2 rDNA.1, Lane 3 rDNA.1 PCR negative control, Lane 4 intergenic-region.1, Lane 5 intergenic-region.1 PCR negative control, Lane 6 intergenic-region.2, Lane 7 intergenic-region.1 PCR negative control, Lane 8 intergenic-region.2, Lane 9 intergenic-region.2 PCR negative control. Click here for file

Additional file 10 **Full multiple sequence alignment for 18S tick and fly species and 16S *E. coli *units**. Full 18S unit multiple sequence alignment for: 5 tick species *A. americanum, A. glauerti, A. variegatum, A. tuberculatum, A. maculatum *and *R. microplus*; 2 fly species *D. simulans, D. melanogaster*; tick host *B. taurus *and *E. coli *16S. In *E. coli *16S protein binding sites are highlighted S7_S9_S19 complex (red), S8_S15_S17 complex (green), S8_S17 complex (aqua) (Weiner et al 1988). Click here for file
