# Supplementary figures and images for: The complexity of Rhipicephalus (Boophilus) microplus genome characterised through detailed analysis of two BAC clones
Source: BMC Res Notes. 2011 Jul 22;4:254. doi: 10.1186/1756-0500-4-254 (PMC3160391; doi:10.1186/1756-0500-4-254)

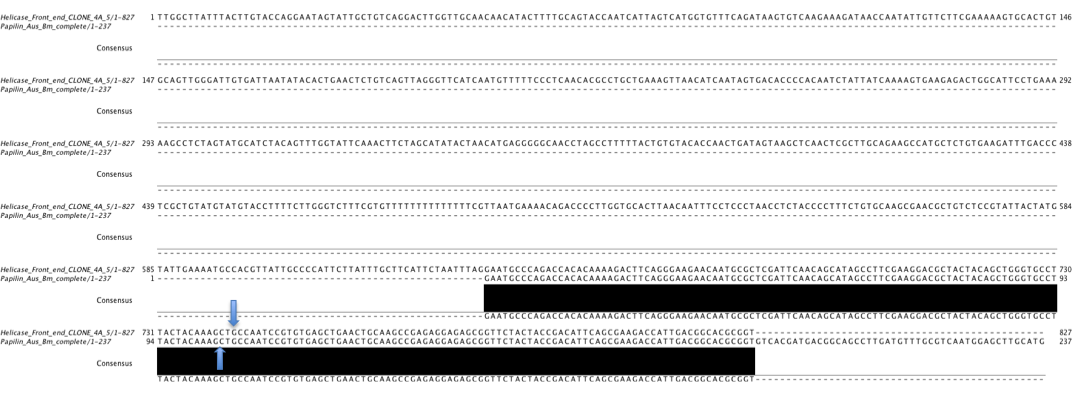

Supplement: Additional file 3 — Sequence alignment of exon overlap between papilin and helicase. Sequence alignment of exon overlap between papilin position 502-738 bp and helicase 3 positions 974-4802 bp. The consensus black bar indicates the region of overlap. Blue arrows indicate exon junctions, papilin T-G positions 604 and 605 and helicase G-C positions 4715, 4716. [file 1756-0500-4-254-S3.PNG]

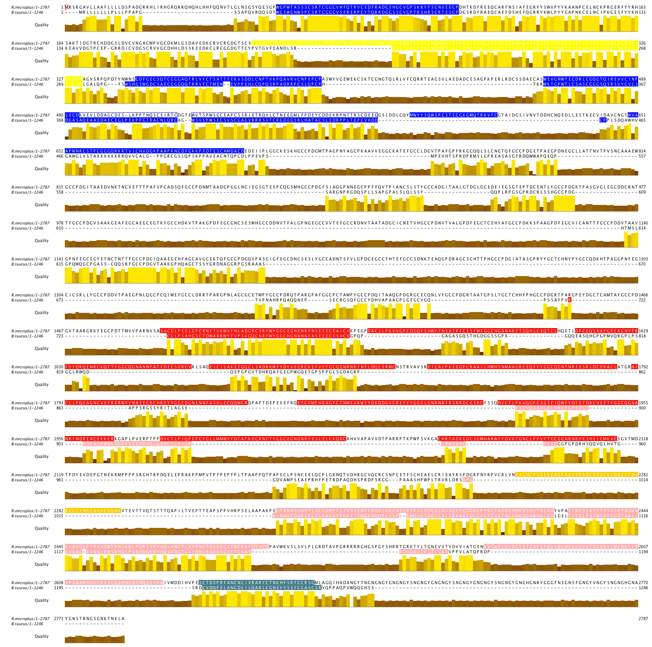

Supplement: Additional file 4 — Rmi and Bos taurus papilin protein sequence alignment. Rmi and Bos taurus papilin protein sequence alignment, R. microplus and B. taurus. Domains are highlighted Kunitz BPTI (red), ADAM spacer1 (yellow), Ig-set (pink), PLAC (purple), WAP (orange) and TSP1 (blue). [file 1756-0500-4-254-S4.PNG]

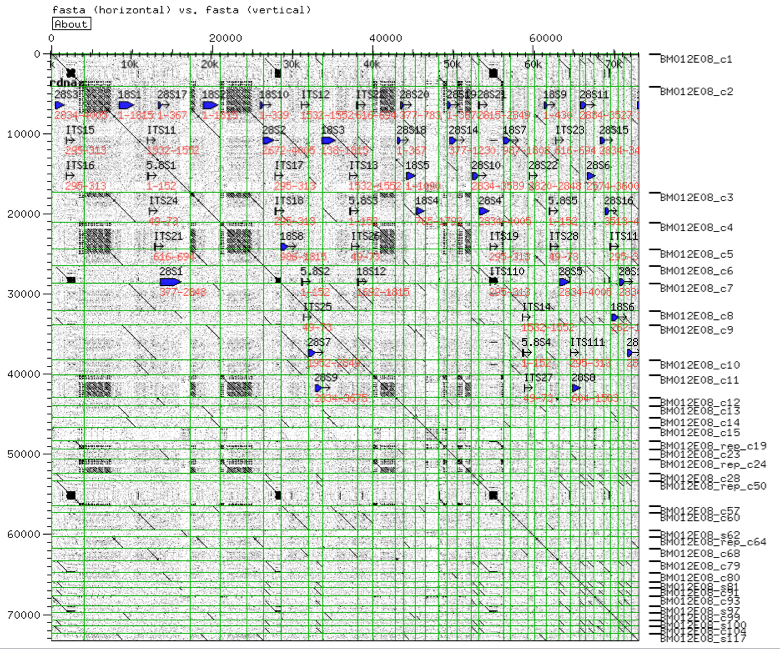

Supplement: Additional file 7 — BM-012-E08 sequence dot matrix with Ambylomma rRNA alignment. Figure of full dot matrix BM-012-E08 with Ambylomma rRNA (blue) alignment. [file 1756-0500-4-254-S7.PNG]

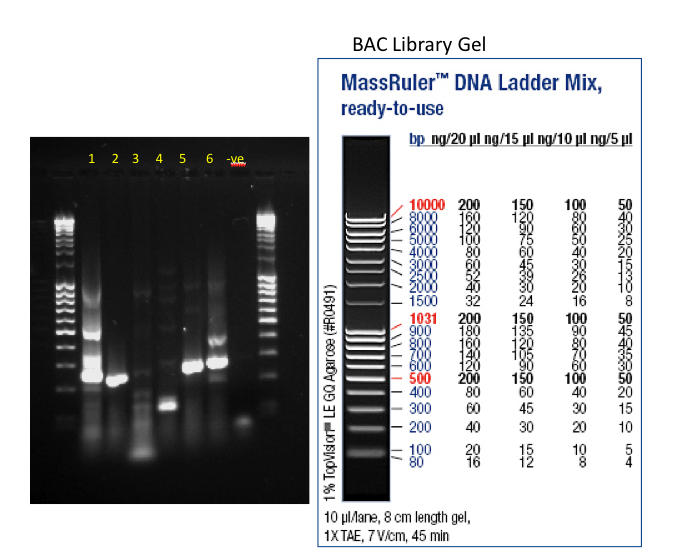

Supplement: Additional file 8 — BM-012-E08 repetitive elements PCR results. PCR results for BM-012-E08 repetitive elements, lanes: 1) 22900 (F1/R1) 2) 22900 (F2/R2) 3) 17000 (F1/R1) 4) 17000 (F2/R2) 5) 38000 (F1/F2) 6) 38000 (F2/R2) 7) negative control. [file 1756-0500-4-254-S8.PNG]

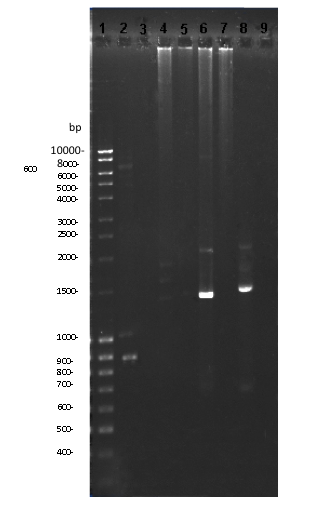

Supplement: Additional file 9 — BM-012-E08 long primer sets to amplify tick genomic DNA. Long primer sets used to amplify tick genomic DNA using Roche Expand Long Template PCR system, Lane 1 Fermentas Mass ruler 80 bp-10 kb (#SM0403), Lane 2 rDNA.1, Lane 3 rDNA.1 PCR negative control, Lane 4 intergenic-region.1, Lane 5 intergenic-region.1 PCR negative control, Lane 6 intergenic-region.2, Lane 7 intergenic-region.1 PCR negative control, Lane 8 intergenic-region.2, Lane 9 intergenic-region.2 PCR negative control. [file 1756-0500-4-254-S9.PNG]

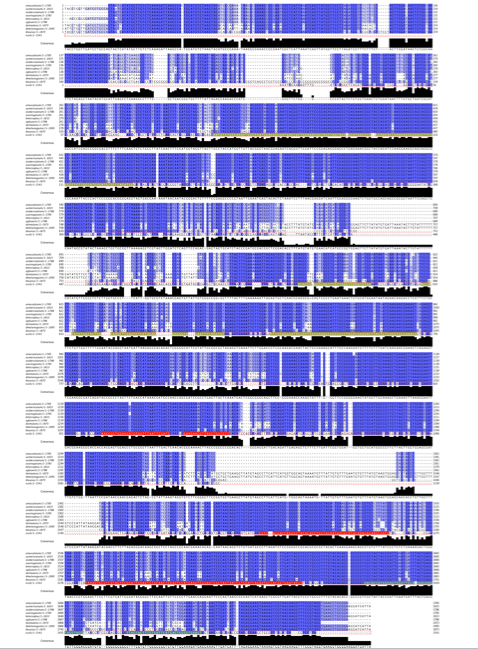

Supplement: Additional file 10 — Full multiple sequence alignment for 18S tick and fly species and 16S E. coli units. Full 18S unit multiple sequence alignment for: 5 tick species A. americanum, A. glauerti, A. variegatum, A. tuberculatum, A. maculatum and R. microplus; 2 fly species D. simulans, D. melanogaster; tick host B. taurus and E. coli 16S. In E. coli 16S protein binding sites are highlighted S7_S9_S19 complex (red), S8_S15_S17 complex (green), S8_S17 complex (aqua) (Weiner et al 1988). [file 1756-0500-4-254-S10.PNG]
